# QC-Chain: Fast and Holistic Quality Control Method for Next-Generation Sequencing Data

**DOI:** 10.1371/journal.pone.0060234

**Published:** 2013-04-02

**Authors:** Qian Zhou, Xiaoquan Su, Anhui Wang, Jian Xu, Kang Ning

**Affiliations:** 1 CAS Key Laboratory of Biofuels and Shandong Key Laboratory of Energy Genetics, Qingdao Institute of Bioenergy and Bioprocess Technology, Chinese Academy of Sciences, Qingdao, Shandong, China; 2 College of Computer and Information Technology, China Three Gorges University, Yichang, Hubei, China; Beijing Institute of Genomics, Chinese Academy of Sciences, China

## Abstract

Next-generation sequencing (NGS) technologies have been widely used in life sciences. However, several kinds of sequencing artifacts, including low-quality reads and contaminating reads, were found to be quite common in raw sequencing data, which compromise downstream analysis. Therefore, quality control (QC) is essential for raw NGS data. However, although a few NGS data quality control tools are publicly available, there are two limitations: First, the processing speed could not cope with the rapid increase of large data volume. Second, with respect to removing the contaminating reads, none of them could identify contaminating sources *de novo*, and they rely heavily on prior information of the contaminating species, which is usually not available in advance. Here we report QC-Chain, a fast, accurate and holistic NGS data quality-control method. The tool synergeticly comprised of user-friendly tools for (1) quality assessment and trimming of raw reads using Parallel-QC, a fast read processing tool; (2) identification, quantification and filtration of unknown contamination to get high-quality clean reads. It was optimized based on parallel computation, so the processing speed is significantly higher than other QC methods. Experiments on simulated and real NGS data have shown that reads with low sequencing quality could be identified and filtered. Possible contaminating sources could be identified and quantified *de novo*, accurately and quickly. Comparison between raw reads and processed reads also showed that subsequent analyses (genome assembly, gene prediction, gene annotation, etc.) results based on processed reads improved significantly in completeness and accuracy. As regard to processing speed, QC-Chain achieves 7–8 time speed-up based on parallel computation as compared to traditional methods. Therefore, QC-Chain is a fast and useful quality control tool for read quality process and *de novo* contamination filtration of NGS reads, which could significantly facilitate downstream analysis. QC-Chain is publicly available at: http://www.computationalbioenergy.org/qc-chain.html.

## Introduction

Next-generation sequencing (NGS) technologies, which could produce numerous sequences (reads) in a single experiment in a relatively short time, have been widely applied in life sciences. However, several kinds of sequencing artifacts, which could introduce serious negative impact on downstream analyses, commonly exist in raw reads, regardless of the sequencing platform. Generally, these sequence artifacts could be classified into two groups:

1. Low sequencing-quality reads, including low quality bases/reads, duplicates, tag sequences, etc. In NGS technology, the qualities of bases on most sequencing platforms will degrade as the run progresses, so it is common to see the quality of base calls falling towards the end of a read. Other kinds of low-quality reads, such as duplicates and tag sequences (such as adaptor and barcode) are introduced by PCR amplification bias and errors during library construction. Although rigorous quality filtration and trimming on Illumina data may remove a large proportion of the reads, it greatly improves the accuracy of subsequent analysis results [1]. For the sequencing quality problem, other than the QC pipeline supplied by the sequencing instrument manufactures, a few online/standalone tools are publicly available, such as PRINSEQ [2], FASTX-Toolkit (http://hannonlab.cshl.edu/fastx_toolkit/) and NGS-QC Toolkit [3]. These tools have specific features and were developed based on different concepts and algorithms, yet are not sufficiently optimized on their own.
2. Contaminating reads from known and unknown species other than sequencing target. The contamination in the sequencing dataset is also of frequent occurrence, which can be caused by artificial experiment fault during the sample preparation, library construction and other experiment steps. Besides, the DNA/RNA sample itself may contain some nucleotides from unexpected species, which are hard to be excluded by biological experiments. For example, we purified the cells of several algae species for genomic sequencing in our laboratory, but still detected various bacteria sequences, although in low amount (1%–5%), in its NGS dataset (unpublished data). The existence of these contaminating reads will affect the downstream analyses seriously and may lead to erroneous results. However, since the contamination situation is complex and divergent for different samples, few strategies and specialized tools are available to tell the users whether there is any contamination (or if yes, what the contaminations are) in a comprehensive, quick and precise way. Consequently, the assessment and removal of the undesirable reads can become highly difficult. Currently, most of the published methods are based on identification and removal of known contaminating sources by optimized alignment method, such as DeconSeq [4] or common alignment software, such as Bowtie [5]. However, most of them rely heavily on known information of the possible contaminating species, but are very limited if the contaminating species is unknown.

Currently, few QC tools provide fast and holistic solutions for both of the above two quality control problems on NGS data. As an essential first step before downstream analyses of NGS data, the QC approach should be able to detect and process both low sequencing-quality reads and contaminating reads.

Another concern of QC processing is the speed. Since the NGS data is usually as huge as up to tens of gigabases, the data processing is both data- and computation- intensive, which requires extensive computational power. Most of the current NGS data QC tools were designed to be used with only single thread, which could not meet the computational requirements of the rapidly increasing number of large-scale sequencing projects. Therefore, holistic and high-performance computational methods are needed for efficient QC analyses.

In this study, we developed the QC-Chain program for NGS data quality assessment and filtration. This program is comprised of two synergetic parts: First, a read quality processing tool, Parallel-QC, was used to perform a diverse reads trimming and filtration process. Second, contaminating reads are qualitatively and quantitatively analyzed and identified by the “rDNA-reads based” and “random-reads based” methods, respectively, and then filtered by alignment tools. Furthermore, to evaluate the quality of reads after trimming and contamination filtration, downstream analyses, including genome assembly, gene model prediction and functional annotation, were performed based on raw and clean reads, respectively. Results showed that through QC-Chain, high-quality clean reads could be obtained with high efficiency, which can serve for subsequent analyses. This QC system could be applied to NGS data in FASTQ or FASTA format produced by Illumina, 454 and other sequencing platforms for genomic and metagenomic sequencing experiments. Furthermore, the speed of QC-Chain can be very fast because it is optimized by parallel computation. QC-Chain is publicly available at: http://www.computationalbioenergy.org/qc-chain.html.

## Methods

### The Overall Quality Control Strategy

**Figure 1:**
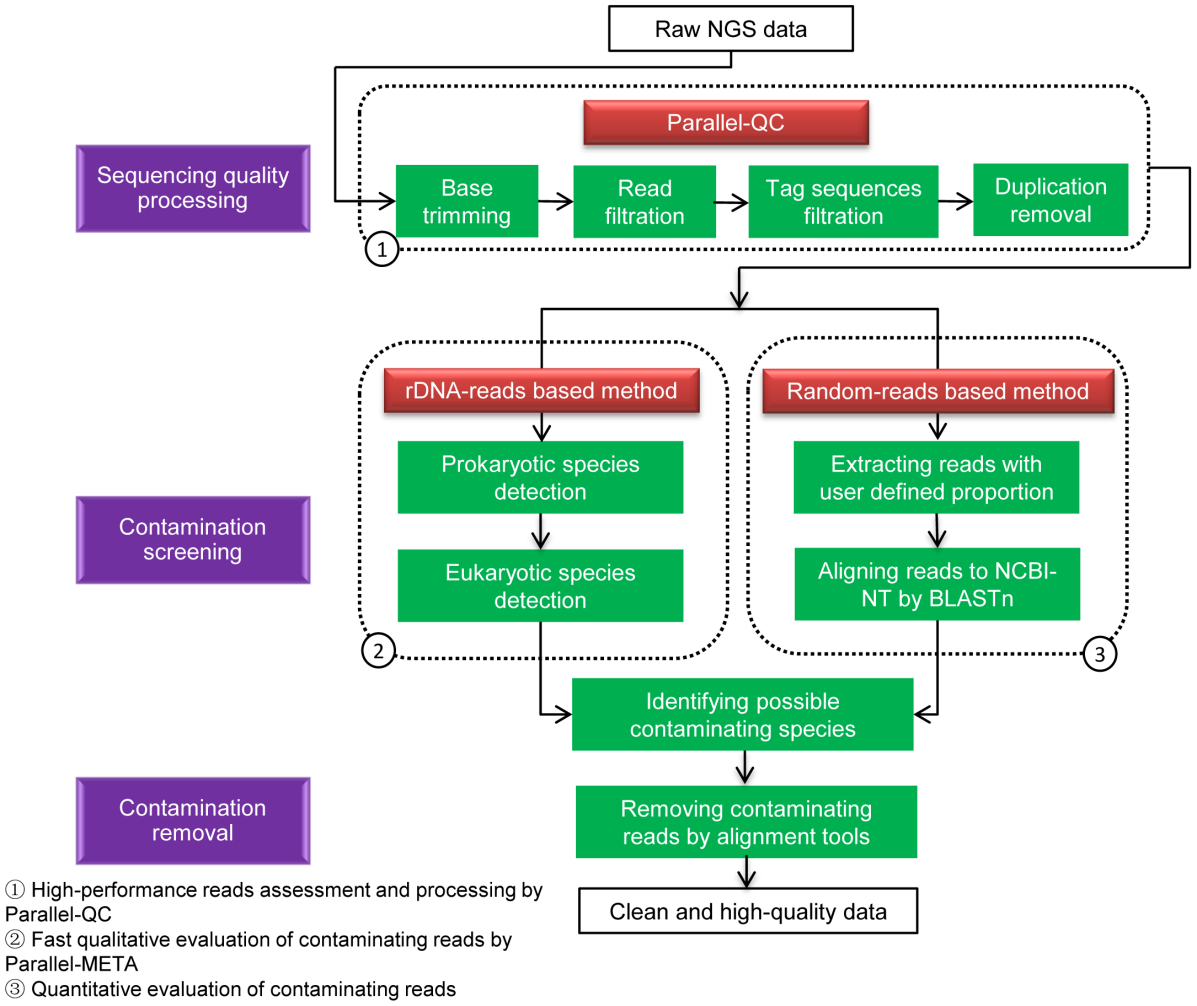
The overall workflow of QC-Chain.

The objectives of QC-Chain include (1) retrieving reads with high quality; (2) identifying and quantifying the source of contaminations, and filtering contaminating reads; (3) accomplishing the QC process in a relatively short time. To achieve these objectives, the overall method of QC-Chain includes sequencing quality assessment and trimming, and contamination screening and removal. Additionally, evaluation and comparison of downstream analysis results using reads after QC were also included as an important component for this holistic approach. The strategy and workflow of the method is shown in **Figure 1**, with detailed procedures as described below.

### Read Quality Assessment and Trimming by Parallel-QC

The sequencing quality assessment and trimming is the first step for NGS data quality control, which requires both accuracy and efficiency. To accomplish this step, we developed a parallel quality control software, Parallel-QC, which could be used to trim, filter and remove low sequencing-quality reads from NGS data. Parallel-QC is developed by Linux C++ and multi-thread technology based on multi-core X86 CPU platform, and is compatible for X86 and X86-64 Linux.

Specifically, by Parallel-QC, sequences could be trimmed to a specific length; low-quality bases within reads could be trimmed from both 5′ and 3′ ends; low-quality reads could be filtered by quality value with user defined percentage; duplications could be identified and removed. For tag sequences filtration, multiple tag sequences could be aligned and shifted on both 5' and 3' ends of the reads with mismatches allowed, and the positive aligned reads could be removed.

To significantly accelerate the speed of computation, Parallel-QC parallelizes the sequencing quality evaluation and filtration steps by assigning balanced and weighted tasks to independent threads, which could be executed on different CPU cores simultaneously. In addition, all progresses could be completed with only one disk I/O operation, which highly improves the efficiency of analysis. On the other hand, the multiple steps can be accomplished by using a single command line with user-friendly options. Therefore, Parallel-QC significantly shortens the processing time compared to traditional single core CPU based method, and simplifies user’s operation compared to using multiple single function QC tools.

### Identification and Removal of Contaminating Reads

The aim of contamination screening is to identify and quantify the (mostly unknown) source of contaminations, filter the contaminating reads, and obtain the processed reads as clean as possible. We adopted two complementary strategies, both of which could provide (known and unknown) species information of the dataset.

In the “rDNA-reads based” method, ribosomal DNA reads were used to qualitatively detect the taxonomical structures of the dataset quickly. Ribosomal RNAs, such as 16S (for prokaryote) and 18S (for eukaryotes) sequences, are good indicators to characterize prokaryotic and eukaryotic species and are commonly used in phylogenetic analysis. They are also widely used in metagenomic analysis to detect the community structure. Here we applied Parallel-META [6], a high-performance 16S/18S rRNA analysis pipeline to report the taxonomic classification, construction and distribution of NGS reads. Parallel-META is a GPU and Multi-Core CPU based software, which firstly extracts the (user selected) 16S or 18S rRNA sequences from the input data and aligns the obtained rRNA reads to several optional databases, including RDP [7], GREENGENES [8] and SILVA [9]. The taxonomy information is produced and then shown in a dynamic graphic view with corresponding species’ proportion. Additionally, in QC-Chain, Parallel-META was updated to be able to accomplish eukaryotic species screening and identification, but in previous version it could only identify prokaryotic species. Through this approach, all the possible species sources of the raw reads, including both prokaryotic and eukaryotic information, could be detected *de novo*.

The other method is “random-reads based”, which could quantitatively provide the species information. Generally, detecting all possible contaminations requires aligning reads to a comprehensive database, which includes species records as many as possible. The most popular and widely-used alignment method is BLAST against NCBI (National Center for Biotechnology Information) database (http://www.ncbi.nlm.nih.gov/). However, it is known that BLAST is a time-consuming process and the speed is a bottleneck, especially when analyzing immense amount of reads. An alternative is to reduce the size of the query data and perform BLAST to get the species information quickly. With such a consideration, we developed an in-house script which could randomly extract reads from the raw reads with a user-defined proportion of all reads. The extracted reads were then aligned to NCBI-NT database using BLASTn, to extract species information in a relatively short time.

The above two approaches are complementary and synergetic to each other: rDNA-reads based method could quickly screen and identify the possible contaminating species. The random-reads based method could provide quantitative evaluation of the contaminations, and also help to verify the result of rDNA-reads based method.

After confirming the contaminating sources by combining the results of the above two methods, the contaminating reads are filtered by the alignment tool Bowtie 0.12.8 [5] with default parameters: reads aligned to contaminating species’ genomes are filtered out.

### Assessment of the Overall QC Results by Downstream Analysis

To evaluate the effect of QC-Chain, downstream analyses were performed to assess and compare the results obtained from reads both pre-QC and after-QC, respectively. In the following parts of this work, the combined original pre-QC reads were referred to as “total reads”, the reads passed the contamination screening were referred to as “clean reads”, and the reads coming from the target genomic or metagenomic sources were referred to as “control reads”.

For the simulated genomic data, the genome assembly was performed by Velvet 1.2.03 [10] on total reads, clean reads and control reads, respectively. The parameters used were: “-exp_cov 70, -cov_cutoff 4, -ins_length 500” and others are set as default. Several indexes, including number of contigs, N50 size and assembly size were considered to evaluate the analysis result. Augustus 2.5.5 [11] was used to predict the open reading frames (ORFs) from the assembly result. The protein sequences of the reference genome were used as the reference to test the accuracy of the gene structure predicted. Specifically, the protein sequences predicted from the assembly of total reads, clean reads and control reads were aligned to reference proteins by BLASTp, respectively and the false positive rate (FPR) were calculated as:



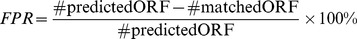
.

For the simulated metagenomic data, *de novo* metagenome assembly and functional analysis were performed with total reads, clean reads and control reads, respectively. Each dataset was firstly assembled using IDBA_UD [12], based on which ORFs were predicted by MetaGeneMark [13]. Simultaneously, those contigs with more than 50 bp in length were submitted to MG-RAST (http://metagenomics.anl.gov) for organism abundance analysis and functional gene annotation. GC distribution and rarefaction curves were generated from MG-RAST automatically. Functional analysis was performed by aligning the predicted ORFs of each dataset to COG database using a maximum e-value of 1e−5 and a minimum identity of 60%. Differences were calculated using one-tailed paired *t-test*, with asterisks denoting statistical significance (NS: not significant; *: *p*<0.05; **: *p*<0.01).

## Results and Discussion

### Reference Genomes and Datasets Used

Both simulated and real NGS data were used in this study. To evaluate the performance of QC-Chain, genomes of multiple species (prokaryotic, eukaryotic with model and non-model species) were used, based on which the simulated datasets for genomic and metagenomic data were generated. On the other hand, since the simulated data is designed as of relatively high quality and free of tag sequences, real NGS data was used to test the performance of sequencing quality evaluation and trimming by Parallel-QC.

To obtain simulated reads, reference genomes were downloaded from NCBI (http://www.ncbi.nlm.nih.gov/). The program DWGSIM 0.1.8 (https://github.com/nh13/DWGSIM) was used to generate simulated sequences from the reference genomes. Specifically, (1) the simulated genomic data contains reads from *Saccharomyces cerevisiae*, representing eukaryotic model species. A certain proportions of simulated bacterial reads from *Clostridium thermocellum* and *Escherichia coli* were included as contaminating sequences to simulate the possible wet-lab contaminations (**Table 1**). Pair-ended sequences were created with an insert-size of 500 bp between two ends, an average error rate of 1% and read length of 100 bp. The resulting simulated data contained 10,856,948 reads, with a total size of 1.08 Gbp; (2) the simulated metagenomic data included simulated reads from 10 oral microbial genomes (including *Actinomyces naeslundii* MG1,*Fusobacterium nucleatum* ATCC 25586,*Haemophilus parainfluenzae* ATCC 33392,*Neisseria elongate* ATCC 29315,*Porphyromonas gingivalis* W83,*Rothia aeria* F0474,*Streptococcus mitis* NCTC 12261,*Streptococcus mutans* UA 159,*Streptococcus oralis* ATCC 35037 and *Streptococcus sanguinis* SK36), which were downloaded from Human Oral Microbiome Database (http://www.homd.org/). Reads generated from *Homo sapiens* and *Chlamydomonas reinhardtii* genomes were used to simulate the contaminating sequences that could possibly come from wet-lab contaminations (*C. reinhardtii*) and sample host (human) (**Table 1**).

**Table 1:** Summary of the simulated data used in this study.

Dataset	Species	Genome coverage	# Reads	Reads%	Read length	Size (Mb)
	***Saccharomyces cerevisiae***	70.0	8,957,552	82.5	100	895.7
**Genomic**	***Clostridium thermocellum***	20.0	726,328	6.7	100	72.6
	***E. coli***	20.0	1,173,068	10.8	100	117.3
	**Total**	N/A	10,856,948	100	100	1,085.7
	***Homo sapiens***	0.3	13,965,464	50.5	70	977.6
**Metagenomic**	***Chlamydomonas reinhardtii***	2.0	3,377,606	12.2	70	236.4
	**10 Oral microbial genomes (see results and discussion)**	30.0	10,306,092	37.3	70	721.4
	**Total**	N/A	27,649,162	100	70	1,935.4

For real NGS data, genomic DNA of two human saliva samples (S1 and S2) and two in-house sequenced algae (Scenedesmus) samples (A1 and A2) were used to construct pair-ended libraries, respectively. They were then sequenced by Illumina GAIIx system (Illumina, San Diego, CA, USA), with an insert size of 400 bp and read length of 75 bp (human saliva) and 100 bp (algae), respectively (**Table 2**). These four real sequencing datasets were used as input to test the performance of Parallel-QC, and all sequences could be downloaded from webpage of QC-Chain (http://computationalbioenergy.org/qc-chain.html).

**Table 2:** Statistics of real NGS data as testing data for Parallel-QC.

Sample	Raw reads		Processed reads	
	Size (Mb)	# Reads	Size (Mb)	# Reads
**S1**	321.8	4,233,904	88.0	1,353,262
**S2**	7,290.7	9,592,980	305.0	4,693,064
**A1**	1,544.9	15,449,564	150,507.9	1,505,079
**A2**	1,707.4	17,074,757	79,966.4	799,664

S1, S2: human saliva DNA samples.

A1, A2: in-house sequenced algae DNA samples.

### Processing of Low Sequencing-quality Reads

**Figure 2:**
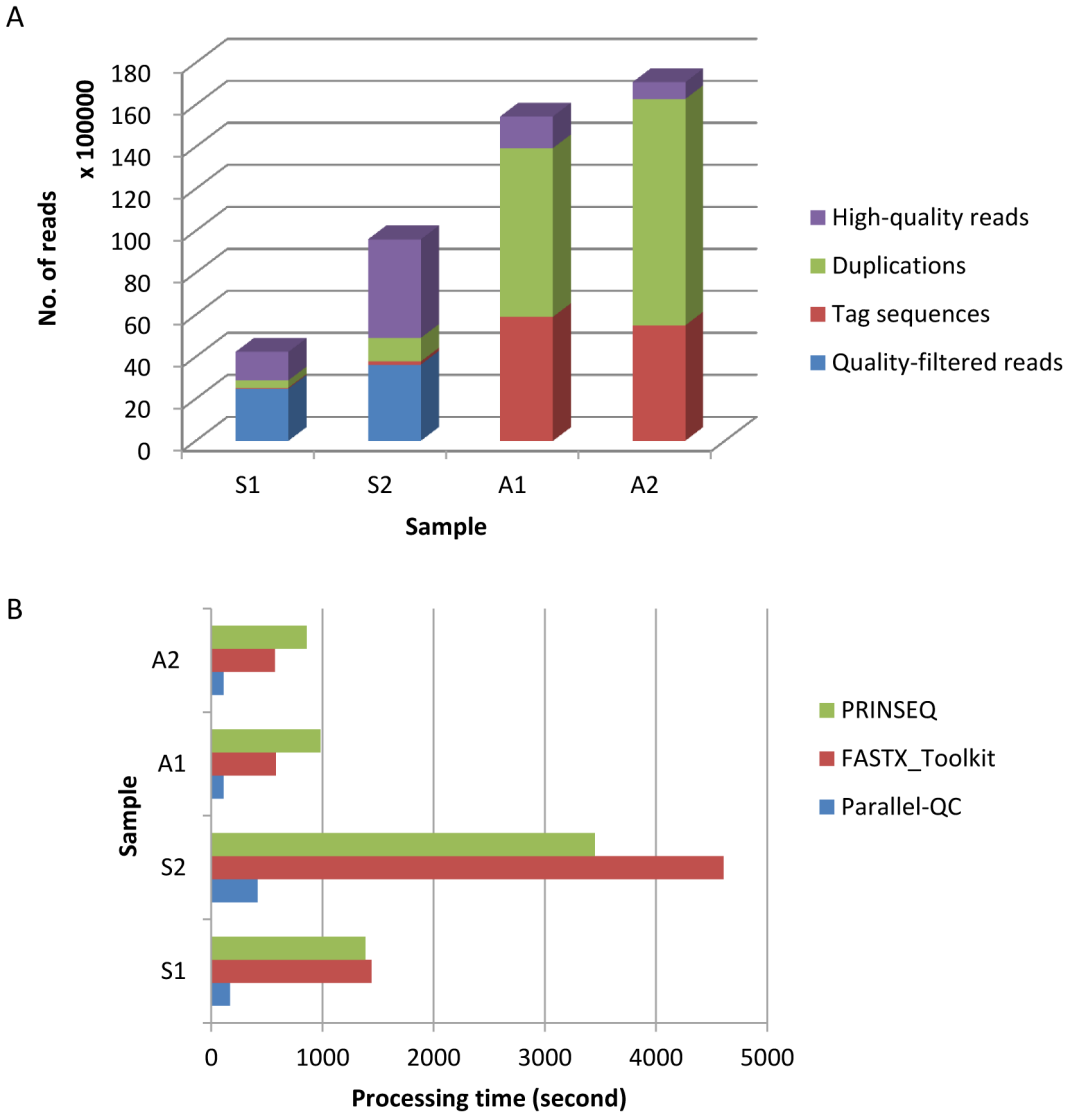
Evaluation of read quality on real NGS data by QC-Chain using Parallel-QC. (A) Summary of sequencing-quality evaluation. (B) Comparison of running time of Parallel-QC, FASTX_Toolkit and PRINSEQ. S1, S2: human saliva DNA samples; A1, A2: in-house sequenced algae DNA samples. All the sequences could be downloadable from http://computationalbioenergy.org/qc-chain.html.

Real NGS datasets were used to test the performance of Parallel-QC for read quality processing, because real data includes various sequencing artifacts, which could be trimmed, filtered and removed by Parallel-QC. Yet as we don’t know the read distribution situation of real data, even if we can use QC-Chain to identify the contaminations, it is difficult to evaluate the accuracy of the method. Therefore, real data was not used for contamination identification. For read quality processing, using quality score ≥20 as a threshold, the read length of two human saliva datasets (S1 and S2) were trimmed to 65 bp and 32% and 49% of the raw reads passed the quality checking; for the two algal genome samples (A1 and A2), the final retained read length was 100 bp and only 10% and 5% of the raw reads were kept as good reads (**Figure 2A**). In these case studies, the size of processed reads is significantly smaller than that of raw reads (**Table 2** **)**, from which it could be anticipated that the subsequent analysis will be compromised significantly if the low quality reads were not removed.

To compare the speed of read processing by different tools, the running time of program FASTX_Toolkit, PRINSEQ and Parallel-QC were compared based on the four testing datasets (A1, A2, S1, S2). These tests were performed on a computational server, which has 2 Intel Xeon X5650, 2.66 GHz clock with 12 cores in total, 72 GB DDR3 ECC RAM, 3TB 7200 RPM HDD (no RAID) and the running time on each dataset was compared. To reduce the effect of system-wise randomness and noises on the results, each input data was analyzed three times and the average results were provided for comparison. A speed-up of an average 7.5 and 8.3 times have been achieved using Parallel-QC compare to FASTX_Toolkit and PRINSEQ, respectively (**Figure 2B**).

With respect to the read-quality processing, a few toolkit/software packages have been developed and made publicly available. Each of them has different features: to name a few examples, FASTX_Toolkit is a collection of command line tools for NGS data preprocessing. However, since it accomplishes diverse procedures of reads processing separately and depends on user’s input for every step, it is time-consuming and complex to use. PRINSEQ is able to process the reads easily and comprehensively, however, our results showed that its processing speed is not satisfactorily fast. In contrast, Parallel-QC, which employs parallel technology and combines multiple optimizations for data processing, could improve the efficiency significantly.

### Recap: Key Features of Parallel-QC

#### Parallel and fast processing

Most of the processing steps executed by Parallel-QC were accomplished using parallel computation, including base-trimming, quality-filtration, screening and removal of tag sequences and duplications. A parameter (-t) is available for defining the number of CPU cores to be used, and if multiple CPU cores are required, the read processing procedures will be processed in parallel on different CPU cores simultaneously. Therefore, the processing speed could be improved significantly. Another reason for the fast running of Parallel-QC is that the whole processing could be completed with only one disc I/O processing, which is highly efficient, especially when processing very large datasets.

#### Multiple tag sequences processing

In Parallel-QC, multiple tag sequences could be detected and filtered within a single run. All the standard tag sequences used in Illumina and Roche 454 sequencing platforms could be filtered by default setting. Alternatively, users could use specified sequence files (in FASTA format) containing candidate sequences to tell Parallel-QC about the filtration targets.

#### Pair-ended reads processing

It is important to maintain the pairing information in the processing progress, since the pair-ended relationship of reads is essential to many of the subsequent analysis, such as alignment and genome assembly. Parallel-QC provides an option (-k, refers to website for details) to keep the paired reads in the output or not. It is able to check the quality of both ends of the paired reads simultaneously in every processing procedure and export the filtered corresponding paired reads, which cannot be realized by most of other available QC tools.

#### Summary report

A clear summary report is supplied by Parallel-QC, showing the information of input reads, parameters used, output of each processing step and final result, which provides a clear overview of the reads status before and after process.

### Contamination Screening and Removal

Simulated data is designed with relatively high read quality, so it is not good testing dataset for read quality processing. On the other hand, they are good models for contamination identification since we are clear about the contamination species and read distribution. Therefore, simulated genomic and metagenomic data were used to test the performance of contamination identification of QC-Chain.

#### Contaminating reads identification

**Figure 3:**
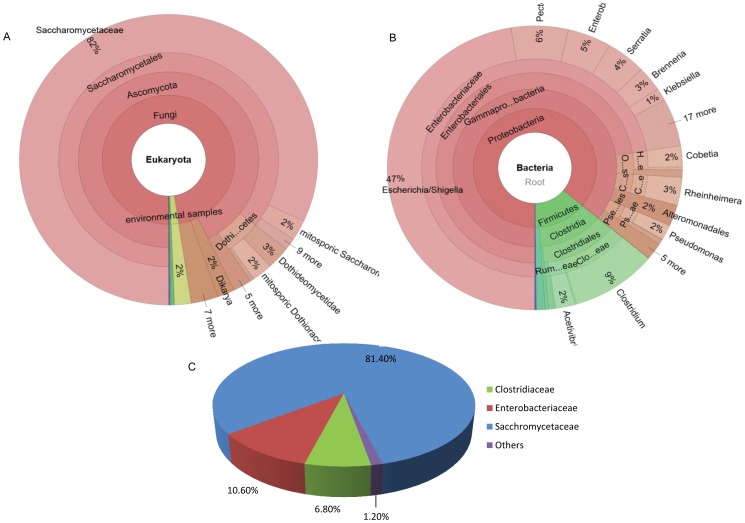
Possible source species identified from simulated genomic data by rDNA-reads based method of QC-Chain. (A) 18S reads distribution identified by rDNA-reads based method. (B) 16S reads distribution identified by rDNA-reads based method. (C) Quantitative distribution of the reads identified by random-reads based method.

For the simulated genomic data, using rDNA-reads based method, 18S and 16S rDNA sequences could be identified and characterized to detect eukaryotic and prokaryotic organisms, respectively. Extracted 18S sequences dominantly mapped to Saccharomycetaceae, and the remaining ones were distributed in a variety of other species (**Figure 3A**). Identified 16S rDNA reads exhibited abundant positive alignments to Clostridiaceae and Enterobacteriaceaeare, while others matched to diverse species each with a proportion lower than 2% (**Figure 3B**). This result suggested that other than Saccharomycetaceae, a relatively large number (but their relative quantity not clear) of Clostridiaceae and Enterobacteriaceae reads are included in the sample. Considering the sequence conservations in the rDNA sequences among different species, only the dominant species should be considered as the possible contamination.

When using the random-reads based method, 0.1% of the total reads were randomly extracted and aligned to NCBI-nt using BLASTn. A total of 10,243 reads got sequence homology in the database, among which 6.8% had best hit to *C. thermocellum*, 10.6% were mapped to *E. coli*, and 81.4% were aligned to *S. cerevisiae*, with the Kullback-Leibler divergence estimate of 0.056 from true-reads distribution (**Figure 3C**). These results coincided with that of the rDNA-reads based method. Moreover, the read distribution among species is highly consistent with the taxonomical distribution of the simulated data (**Table 1**). Thus the random-reads based method is suitable for quantitative analysis of contaminating reads.

**Figure 4:**
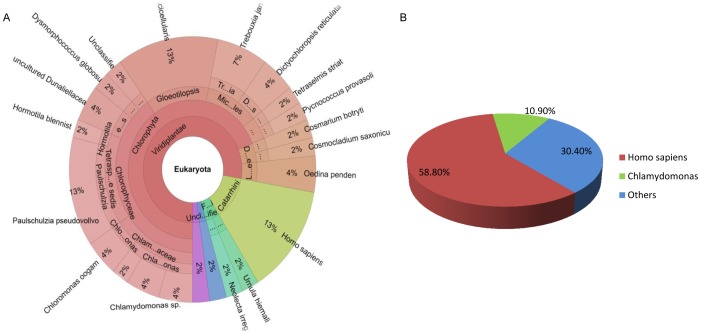
Possible source species identified from simulated metagenomic data by rDNA-reads based method of QC-Chain. (A) 18S reads distribution identified by rDNA-reads based method. (B) Quantitative distribution of the reads identified by random-reads based method.

For the simulated metagenomic data, eukaryotic species were considered as possible contaminations. By rDNA-reads based method, 18S rDNAs (for eukaryote) were identified using Parallel-META, with results showing that abundant rDNA reads were mapped to Chlorophyta and *Homo sapiens* (**Figure 4A**). However, several Family and species were involved in Chlorophyta, including Chlamydomonadaceae, Volvox, Gloeotilopsis, etc., which rises from the high 18S sequence similarity of these algae species. Then, by random-reads based method, 0.1% of the total reads were extracted and the BLAST result showed that 58.8% and 10.9% of the extracted reads were aligned to *Homo sapiens* and Chlamydomonas, respectively. The KL divergence between the reads-source distributions in our results and reference (truth) is 0.014 (**Figure 4B**). The predicted relative quantities of these species are consistent with those from the simulated metagenomic data. Several other eukaryotic species were also identified, which are evolutionally close to Chlamydomonas and human with a few number of reads. These organisms may be matched due to high sequence similarities by BLAST, and therefore, they were probably not contaminating species. Combining the results of the above two methods, Chlamydomonas and human were successfully identified as two dominant contaminating sources in the simulated metagenomic data.

The analyses of both simulated genomic and metagenomic data showed that the two contamination screening methods are complementary and synergetic. By rDNA-reads based method, the possible contaminating species could be quickly identified qualitatively. Then, using random-reads based method, more detailed and quantitative proportion information of contaminating organisms is provided. The two methods could also mutually verify their results.

#### Speed of contamination screening

The amount of data produced by NGS technology is significantly larger than that generated by earlier sequencing techniques and can reach tens of gigabases for a single dataset. Thus, it requires fast and accurate approaches for data processing. Since “rDNA-reads based method” (based on improved Parallel-META) is constructed based on parallelization algorithm, at least 15 times speed-up was achieved compared to traditional CPU-based analysis method, with the same accuracy [6]. In our experiment, it took only 5 min to detect both the eukaryotic and prokaryotic contaminating sources for the simulated genomic data, and 16 min to identify the possible eukaryotic species included in the simulated metagenomic data. Thus, it ensures that the taxonomical screening of huge NGS data is fast and accurate.

For random-reads based method, using 0.1% randomly extracted reads from simulated genomic and metagenomic data, it took 10 hours and 24 hours to finish the BLASTn to NCBI-nt database, respectively, from which it could be estimated that the original BLAST will consume almost a thousand more time. Although it takes more time to finish the analysis by random-reads based method than rDNA-reads based method, since the proportion of aligned reads reflects the relative abundance of each speices, it is suitable for quantitative evaluation of contaminating sources.

#### Contaminating reads removal

Once the contaminating species is revealed, and if the species has a reference genome sequence, the contaminating reads could be filtered using alignment programs. A large number of alignment tools are available, whose performances have been compared and assessed in literatures [14]–[16]. The selection and usage of the alignment tools is not within the scope of this study, and here we used Bowtie 0.12.8 [5] to remove possible contaminating reads (by alignment to contaminating species’ genomes) to obtain the “clean reads”.

### Assessment of Filtered Reads by Holistic Approach

To evaluate the effect of our QC methods, downstream analyses were applied on the simulated data.

For the simulated genomic data, reads mapped to *C. thermocellum* and *E. coli* were removed to produce the ‘clean reads’. Genome assembly was performed with total reads, clean reads and control reads, respectively. It showed that the clean reads achieved a precise assembly size (11.6 Mbp) of the target sequencing species (*S. cerevisiae* with genome size of 12 Mbp, Saccharomyces Genome Database, http://www.yeastgenome.org/), which was consistent to that using the control reads (11.6 Mbp) (**Table 3**). Whereas the assembly size is apparently larger (20.4 Mbp) using total reads, with contaminating reads from bacteria. The number of contigs and N50 size of clean reads and total reads obtained assemblies also differed greatly (**Table 3**). We also tested the contig identities by mutual-BLAST. The result showed that using the contigs of control reads as the reference, 94% of the contigs obtained from clean reads obtained positive alignments, but only 70% of the contigs of total reads obtained positive alignments. All these results indicated that in genome assembly based on total reads, contaminating reads have been misassembled into the contigs and thus have filled the gaps mistakenly in the “total-assembly”.

**Table 3:** Genome assembly and ORF prediction using total, clean and control reads of simulated data, respectively.

Dataset		Assembly size (Mb)	# contigs	N50 (bp)	# ORFpredicted	# ORFs matching reference	FPR*
	**Total reads**	20.4	381	174,181	10,143	6,736	33.6%
**Genomic**	**Clean reads**	11.6	2,693	9,422	6,116	5,617	8.2%
	**Control reads**	11.6	1,517	187,516	5,531	5,484	0.9%
	**Total reads**	24.4	6,989	84,411	24,237	NA	NA
**Metagen-omic**	**Clean reads**	22.0	2,933	69,821	22,774	NA	NA
	**Control reads**	22.3	2,933	96,904	22,307	NA	NA

* FPR: false positive rate of the genes predicted, using *S. cerevisiae* as reference.

Furthermore, 10,143, 6,116 and 5,531 ORFs were predicted using the assemblies of total reads, clean reads and control reads, respectively. The number of ORFs overlapping the reference proteins was calculated and the FPR of clean reads (8.2%) was significantly lower than that of total reads (33.6%), indicating that the ORFs predicted based on clean reads are more accurate than those based on total reads (**Table 3**). The analysis also showed that some differences exist in the analysis results of clean reads and control reads, such as number of contigs, N50 size and number of ORF predicted (**Table 3**). This is because the alignment method did not remove all of the contaminating reads, and a small proportion of contaminating reads were left in the clean reads, which affected the further analysis result.

**Figure 5:**
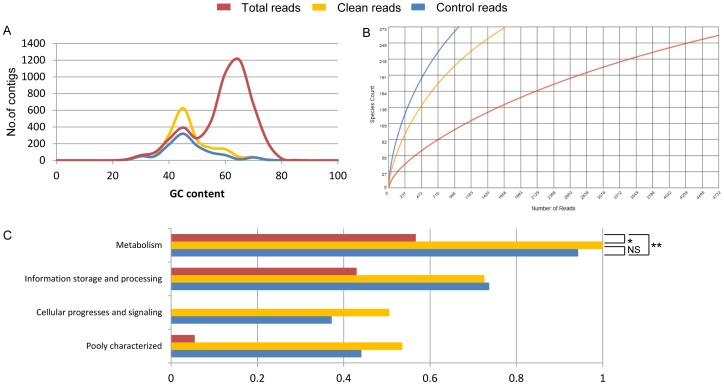
Comparison of the results from clean, total and control reads of simulated metagenomic data. (A) GC distribution pattern. (B) Rarefaction curve that could discriminate the richness of different bacterial species, Y-axies: species count, X-axis: the number of reads. (C) Functional categories based on COG database. Abundance of each category between the three datasets was compared pair-wise (*p<0.05; **p<0.01; NS: not significant).

For the simulated metagenomic data, the assembly and gene prediction results were significantly different, with 6,989 contigs and 24,237 genes obtained from total reads, and 2,933 contigs and 22,307 genes obtained from clean reads, respectively (**Table 3**). The clean reads exhibited a very similar set of statistics to those of control reads (**Table 3**). The GC contents and distribution pattern of clean reads and control reads are very similar, which are distinctly different from that of total reads (**Figure 5A**). The individual rarefaction curves of both clean reads and control reads showed a similar pattern of increasing bacterial diversity, which were different from those of total reads (**Figure 5B**). Remarkably, when 946 sequences were sampled, clean reads and control reads both showed two-fold more bacterial richness than total reads, indicating that the eukaryotic contamination could reduce the richness of bacterial species dramatically (**Figure 5B**). The functional distribution and abundance analysis based on COG database also reported similar relative abundances in the four categories of “metabolism”, “information storage and processing”, “cellular processes and signaling” and “poorly characterized” based on clean and control reads; while those based on total reads are significantly different (**Figure 5C**). Significant differences of the functional abundance were observed between clean reads and total reads (*t-test p*-value = 0.01), as well as between control reads and total reads (*t-test p*-value = 0.002). No significant difference was found between clean reads and control reads (*t-test p*-value = 0.288).

All these analyses and comparisons revealed that the subsequent analytical results based on clean reads and control reads are in high accordance, and remarkably divergent from those of total reads. The significant improvement of the subsequent analysis result using after-QC reads indicated that our QC processes, especially contaminating screening methods, have a significant impact on the quality of the NGS data and benefit the subsequent analysis extensively. It is possible that with the development of more accurate alignment algorithms, improved results in downstream analysis could be expected.

### Comparison with Existing QC Methods

Generally, the QC processing of NGS data should include at least two fundamental aspects: (1) low sequencing-quality reads filtration, and (2) contamination identification and removal. Currently, most of the QC tools are designed specifically to evaluate and filter reads with low sequencing-quality. These tools are developed based on different algorithms and have different features, which may have one or more limitations in different aspects [3]. For example, for the tag sequences removal, the FASTX_Toolkit provides a tool for this purpose, but it depends on user input of the tag sequences once at a time, making the procedure time-consuming and complicated for multiple tag filtrations. PRINSEQ cannot be used to trim the tag sequences directly, but just estimates whether the dataset contains these sequences and checks whether they are removed sufficiently after trimming. Other tools such as CUTADAPT [17] are able to deal with multiple adapters in a single run, but it cannot perform other processing tasks such as duplication filtration. Additionally, the speeds of most of the existing quality filtration tools are not satisfactorily fast. On the other hand, identifying all possible contaminations and removing them require both advanced computational technologies and computational resources. Universal alignment method such as BLAST is extremely time-consuming. Other alignment programs, such as Bowtie [5] and BWA [14], can complete the alignment considerably fast, but rely heavily on the known information of the contaminating species. In our QC strategy, both the read quality filtration and contamination identification/removal are considered and the processing is realized through parallel computation, which could significantly accelerate the quality control process. Comparisons of different features of the NGS QC tools, including QC-Chain, FastQC, FASTX-Toolkit, PRINSEQ, NGS QC Toolkit and DeconSeq are listed in **Table 4**.

**Table 4:** Comparisons of key features of QC-Chain and other QC tools.

Tools	QC-Chain	NGS QCToolkit	FASTX-Toolkit 0.0.13	FastQC 0.10.1	PRINSEQ	DeconSeq
Parallelization	Yes	Yes	No	No	No	No
Pairing information	Yes	Yes	No	No	No	No
Sequencing quality evaluation	Yes	Yes	Yes	Yes	Yes	No
Sequencing quality trimming	Yes	Yes	Yes	No	Yes	No
Duplication trimming	Yes	Yes	Yes	No	Yes	No
Multiple tag sequences filtration	Yes	Yes	No	No	No	No
*De novo* contamination screening	Yes	No	No	No	Yes*	No
Contaminating reads removal	Yes	No	No	No	No	Yes

* Indirect evaluation for metagenomic data.

Taking the simulated genomic data for example, we compared the subsequent analysis results using different tools. When applying QC-Chain on the dataset, the results showed a significant improvement in the downstream analysis (**Table 3**), but when other tools such as FastQC, FASTX-Toolkit, PRINSEQ or NGS QC were applied, since the simulated data were designed to be of high-quality reads, few reads were filtered because of low sequencing-quality and the analysis result is equivalent to that obtained from total reads. On the other hand, none of the above tools (except QC-Chain) contains method to screen and identify possible contaminating reads *ab initio*. When there is no available information of the possible contaminating species, the contaminating reads could not be identified, and the results of DeconSeq (an alignment tool depends on known species) would also be the same as those based on total reads.

### Conclusion

With an aim to design a fast, accurate and holistic solution for the quality control of NGS data, we developed QC-Chain, a method which could identify and filter both low sequencing-quality reads and contaminating reads. Read quality process module (Parallel-QC), together with rRNA identification module (improved Parallel-META) and in-house scripts were used in this method to accomplish the comprehensive quality control process. After trimming and filtering the low sequencing-quality reads by Parallel-QC, possible contaminating sources could be identified and quantified *de novo*, without any *a prior* information of the contamination species. All reads after filtration are then evaluated by a holistic approach, which takes into consideration of down-stream analyses that include genome assembly, gene prediction and annotation, to evaluate whether these reads are of high quality. Additionally, both reads processing and identification of the contaminations are quite fast since they are based on parallel computation.

QC-Chain has been tested on simulated genomic and metagenomic datasets, as well as on some real datasets for some of its modules (read quality assessment and trimming). The results showed that sequencing artifacts, including low quality bases, tag sequences and duplications could be trimmed with high speed. Moreover, contaminating species could be screened and identified qualitatively and quantitatively. Furthermore, downstream analysis results based on the reads passed the QC-Chain system showed significantly higher consistency with control reads, compared to raw reads. Therefore, reads that passed this integrated workflow should be able to serve as high-quality and relatively clean reads for further analysis.

As a general QC method, QC-Chain could be further improved for application on more diverse NGS data, such as methylome sequencing data in which there is a high proportion of biased and duplicated reads [18]. Another possible improvement of current QC-Chain would be higher speed and accuracy, especially on quantitative analysis of both known and unknown contaminations.
